# Supplementary material for: The Systems Biology Research Tool: evolvable open-source software
Source: BMC Syst Biol. 2008 Jun 29;2:55. doi: 10.1186/1752-0509-2-55 (PMC2446383; doi:10.1186/1752-0509-2-55)
Supplement: Additional file 1 — SBRT Archive. An archive of the current version of the Systems Biology Research Tool. [file 1752-0509-2-55-S1.zip › sbrt-1.4.0/doc/developers_guide/api/sbrt/shell/io/fba/SerFbaOptOutputFile.html]

SerFbaOptOutputFile


|  |  |  |
| --- | --- | --- |
| **Overview** \| **Package** \| **Class** \| **Use** \| **Tree** \| **Deprecated** \| **Index** \| **Help** | |  |
| **PREV CLASS**   **NEXT CLASS** | **FRAMES**    **NO FRAMES**     **All Classes** |  |
| SUMMARY: NESTED \| FIELD \| CONSTR \| METHOD | DETAIL: FIELD \| CONSTR \| METHOD |  |


---


## sbrt.shell.io.fba Class SerFbaOptOutputFile

```
java.lang.Object
  sbrt.shell.io.fba.SerFbaOptOutputFile
```

**All Implemented Interfaces:**: ResultsManager<FbaOptComputer.Results>, FbaOptOutputFile, OutputFile<FbaOptComputer.Results>, SbrtFile

---

``` public class SerFbaOptOutputFile extends java.lang.Object implements FbaOptOutputFile ```

This class is used to write output files
containing FBA optimization data as serialized maps.

**Author:**
:   This class was written and documented by
    Jeremiah Wright while in the Wagner lab.

**See Also:**: `SmoFile`

---

**Constructor Summary**

`SerFbaOptOutputFile(java.lang.String fileName,
FileFormat fileFormat,
java.util.Set<java.lang.String> filterKeys,
FbaOptFileLineFormat fbaFormat)`
  
          Constructs a new serialized FBA optimization output file.


| **Method Summary** | |
| --- | --- |
| `void` | `addData(java.util.Map<java.lang.String,java.lang.Double> results)`             Writes the appropriate values in the provided map to this file. |
| `<S extends FbaOptComputer.Results> void` | `addData(S results)`             Writes the appropriate values in the provided `FbaOptimizationComputer.Results` to this file. |
| `void` | `breakPoint()`             Does nothing. |
| `void` | `close()`             Closes this file. |
| `java.lang.String` | `getCanonicalName()`             Returns the canonical name of this file. |
| `FbaOptFileLineFormat` | `getFbaOptFileLineFormat()`             Returns the FBA optimization file line format used to supply a header object for objective values. |
| `java.util.Set<java.lang.String>` | `getFilterKeys()`             Returns the set of keys whose values will be written to this output file when results are added. |
| `java.lang.String` | `getName()`             Returns the canonical name of this file. |
| `java.lang.String` | `getOriginalName()`             Returns the name of this file. |
| `boolean` | `isClosed()`             Indicates if this file has already been closed. |

**Methods inherited from class java.lang.Object**

`clone, equals, finalize, getClass, hashCode, notify, notifyAll, toString, wait, wait, wait`

**Constructor Detail**

### SerFbaOptOutputFile

```
public SerFbaOptOutputFile(java.lang.String fileName,
                           FileFormat fileFormat,
                           java.util.Set<java.lang.String> filterKeys,
                           FbaOptFileLineFormat fbaFormat)
```

:   Constructs a new serialized FBA optimization output file.

    **Parameters:**: `fileName` - the name of the file to create.: `fileFormat` - the format of the file to create.: `filterKeys` - a set of valid keys that will appear in the file. This set can consist of reaction names and the objective function header.: `fbaFormat` - the map format used to supply a header object for objective values.


**Method Detail**

### getFbaOptFileLineFormat

```
public FbaOptFileLineFormat getFbaOptFileLineFormat()
```

:   Returns the FBA optimization file line format used
    to supply a header object for objective values.

    :   **Specified by:**: `getFbaOptFileLineFormat` in interface `FbaOptOutputFile`
    :   **Returns:**: the FBA optimization file line format used to supply a header object for objective values. **See Also:**: `FbaOptFileLineFormat.getObjValueHeader()`

---


### getFilterKeys

```
public java.util.Set<java.lang.String> getFilterKeys()
```

:   Returns the set of keys whose values will be
    written to this output file when results are added.
    This set acts as a type of filter to control which
    values in a set of results are saved, and which are discarded.

    :   **Returns:**: the set of row headers whose values will be written to this output file when a map is added; or `null` if *every* value in an added set of results will be written to this file.

---


### addData

```
public <S extends FbaOptComputer.Results> void addData(S results)
```

:   Writes the appropriate values in the provided
    `FbaOptimizationComputer.Results` to this file.
    The appropriates values are those returned by
    `getFilterKeys()`.

    :   **Specified by:**: `addData` in interface `ResultsManager<FbaOptComputer.Results>`
    :   **Type Parameters:**: `S` - a type extending `FbaOptimizationComputer.Results` **Parameters:**: `results` - the results to write to this file. **Throws:**: `java.lang.IllegalArgumentException` - if the provided results object does not contain all the variables in the set returned by `getFilterKeys()`.: `java.lang.IllegalStateException` - if `close()` has already been called.

---


### addData

```
public void addData(java.util.Map<java.lang.String,java.lang.Double> results)
```

:   Writes the appropriate values in the provided
    map to this file.
    The appropriates values are those returned by
    `getFilterKeys()`.

    :   **Parameters:**: `results` - the results vector to write to this file. **Throws:**: `java.lang.IllegalArgumentException` - if the provided map does not contain all the keys in the set returned by `getFilterKeys()`.: `java.lang.IllegalStateException` - if `close()` has already been called.

---


### close

```
public void close()
```

:   Closes this file. If the file is already closed, no
    action will be taken.

    :   **Specified by:**: `close` in interface `ResultsManager<FbaOptComputer.Results>` **Specified by:**: `close` in interface `SbrtFile`

---


### getName

```
public final java.lang.String getName()
```

:   Returns the canonical name of this file.

    :   **Specified by:**: `getName` in interface `ResultsManager<FbaOptComputer.Results>`
    :   **Returns:**: the canonical name of this file.

---


### getOriginalName

```
public final java.lang.String getOriginalName()
```

:   Returns the name of this file.

    :   **Specified by:**: `getOriginalName` in interface `SbrtFile`
    :   **Returns:**: the name of this file.

---


### getCanonicalName

```
public final java.lang.String getCanonicalName()
```

:   Returns the canonical name of this file.

    :   **Specified by:**: `getCanonicalName` in interface `SbrtFile`
    :   **Returns:**: the canonical name of this file. **See Also:**: `File.getCanonicalPath()`

---


### isClosed

```
public boolean isClosed()
```

:   Indicates if this file has already been closed.

    :   **Specified by:**: `isClosed` in interface `ResultsManager<FbaOptComputer.Results>` **Specified by:**: `isClosed` in interface `SbrtFile`
    :   **Returns:**: `true` if this file is closed; `false` othewise. **See Also:**: `ResultsManager.close()`

---


### breakPoint

```
public void breakPoint()
```

:   Does nothing.

    :   **Specified by:**: `breakPoint` in interface `ResultsManager<FbaOptComputer.Results>`
    :   **Throws:**: `java.lang.IllegalStateException` - if `close()` has already been called.


---


|  |  |  |
| --- | --- | --- |
| **Overview** \| **Package** \| **Class** \| **Use** \| **Tree** \| **Deprecated** \| **Index** \| **Help** | |  |
| **PREV CLASS**   **NEXT CLASS** | **FRAMES**    **NO FRAMES**     **All Classes** |  |
| SUMMARY: NESTED \| FIELD \| CONSTR \| METHOD | DETAIL: FIELD \| CONSTR \| METHOD |  |


---
